# 2-Amino-5-bromo­pyridinium 2-hy­droxy­benzoate

**DOI:** 10.1107/S1600536810029855

**Published:** 2010-07-31

**Authors:** Ching Kheng Quah, Madhukar Hemamalini, Hoong-Kun Fun

**Affiliations:** a X-ray Crystallography Unit, School of Physics, Universiti Sains Malaysia, 11800 USM, Penang, Malaysia

## Abstract

In the title compound, C_5_H_6_BrN_2_^+^·C_7_H_5_O_3_^−^, the 2-amino-5-bromo­pyridinium cation and 2-hy­droxy­benzoate anion are essentially planar with maximum deviations of 0.020 (1) and 0.018 (2) Å, respectively. The anion is stabilized by an intra­molecular O—H⋯O hydrogen bond, which generates an *S*(6) ring motif. In the crystal, the cations and anions are linked by N—H⋯O hydrogen bonds into chains propagating along [010]. The chains contain *R*_2_^2^(8) ring motifs. The structure is further stabilized by π–π stacking inter­actions [centroid–centroid distances = 3.4908 (10) and 3.5927 (10) Å] and also features short Br⋯O contacts [2.9671 (13) Å].

## Related literature

For details of non-covalent inter­actions, see: Remenar *et al.* (2003 ▶); Sokolov *et al.* (2006 ▶). For the importance of salicylic acid, see: Sticher *et al.* (1997 ▶); Rairdan & Delaney (2002 ▶); Nawrath & Métraux (1999 ▶); Wildermuth *et al.* (2001 ▶). For related structures, see: Quah *et al.* (2008 ▶, 2010*a* ▶,*b* ▶). For the stability of the temperature controller used in the data collection, see: Cosier & Glazer (1986 ▶). For bond-length data, see: Allen *et al.* (1987 ▶). For hydrogen-bond motifs, see: Bernstein *et al.* (1995 ▶).
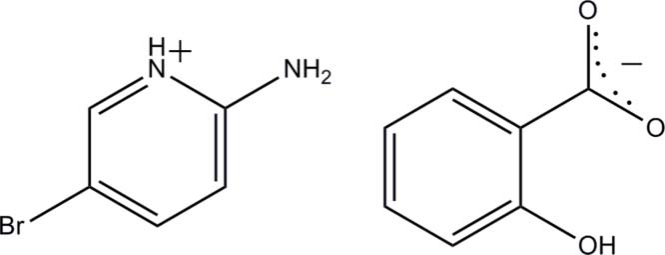


## Experimental

### 

#### Crystal data

C_5_H_6_BrN_2_^+^·C_7_H_5_O_3_^−^
*M* *_r_* = 311.14
Monoclinic, 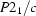

*a* = 8.9498 (2) Å
*b* = 10.8673 (2) Å
*c* = 13.1277 (3) Å
β = 108.704 (1)°
*V* = 1209.37 (4) Å^3^
*Z* = 4
Mo *K*α radiation
μ = 3.40 mm^−1^
*T* = 100 K
0.48 × 0.27 × 0.19 mm

#### Data collection

Bruker SMART APEXII CCD area-detector diffractometer
Absorption correction: multi-scan (*SADABS*; Bruker, 2009 ▶) *T*_min_ = 0.291, *T*_max_ = 0.572
13299 measured reflections
3559 independent reflections
2942 reflections with *I* > 2σ(*I*)
*R*_int_ = 0.023

#### Refinement

*R*[*F* ^2^ > 2σ(*F* ^2^)] = 0.025
*wR*(*F* ^2^) = 0.070
*S* = 1.12
3559 reflections
179 parameters
H atoms treated by a mixture of independent and constrained refinement
Δρ_max_ = 0.62 e Å^−3^
Δρ_min_ = −0.48 e Å^−3^

### 

Data collection: *APEX2* (Bruker, 2009 ▶); cell refinement: *SAINT* (Bruker, 2009 ▶); data reduction: *SAINT*; program(s) used to solve structure: *SHELXTL* (Sheldrick, 2008 ▶); program(s) used to refine structure: *SHELXTL*; molecular graphics: *SHELXTL*; software used to prepare material for publication: *SHELXTL* and *PLATON* (Spek, 2009 ▶).

## Supplementary Material

Crystal structure: contains datablocks global, I. DOI: 10.1107/S1600536810029855/ci5140sup1.cif

Structure factors: contains datablocks I. DOI: 10.1107/S1600536810029855/ci5140Isup2.hkl

Additional supplementary materials:  crystallographic information; 3D view; checkCIF report

## Figures and Tables

**Table 1:** Hydrogen-bond geometry (Å, °)

*D*—H⋯*A*	*D*—H	H⋯*A*	*D*⋯*A*	*D*—H⋯*A*
O1—H1*O*1⋯O2	0.89 (3)	1.66 (3)	2.500 (2)	157 (2)
N1—H1*N*⋯O3	0.96 (2)	1.66 (2)	2.611 (2)	172 (2)
N2—H1*N*2⋯O2	0.85 (3)	1.98 (2)	2.818 (2)	170 (2)
N2—H2*N*2⋯O1^i^	0.82 (2)	2.14 (2)	2.917 (2)	160 (2)

Symmetry code: (i) 
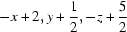
.

**Fig. 1.**
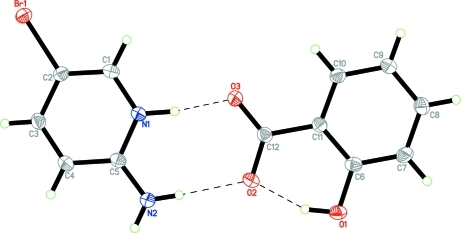
The two ionic units of the title compound, showing 50% probability displacement ellipsoids for non-H atoms and the atom-numbering scheme. Dashed lines indicate hydrogen bonds.

**Fig. 2.**
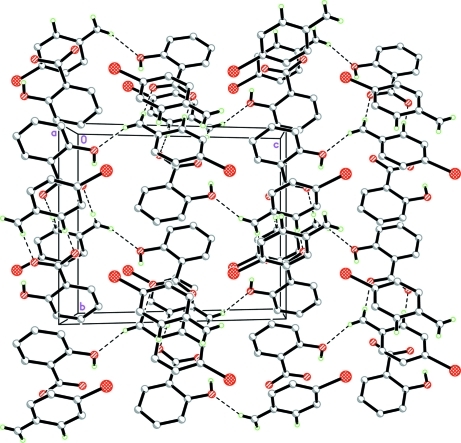
The crystal structure of the title compound, viewed along the a axis. H atoms not involved in hydrogen bonds (dashed lines) have been omitted for clarity.

## supplementary crystallographic information

### Comment

Recently, much attention has been devoted to the design and synthesis of supramolecular architectures assembled *via* various weak noncovalent interactions, such as hydrogen bonds, π···π stacking and C—H···π interactions (Remenar *et al.*, 2003; Sokolov *et al.*, 2006). Salicylic acid (SA) plays a central role in resistance and defense induction in responses from pathogen attacks and also its role in the activation of the hypersensitive response (HR), a form of programmed cell death associated with resistance of plants. Mutants or transgenic plants impaired in the accumulation of SA cannot mount efficient defense responses to pathogens after infection (Sticher *et al.*, 1997). SA depletion by transgenic expression of a bacterial SA hydroxylase encoded by NahG abolishes local and systemic resistance responses to various pathogens (Rairdan & Delaney, 2002). This has been confirmed by the use of Arabidopsis mutants impaired in SA accumulation after pathogen infection (sid1/eds5, sid2), showing higher susceptibility to fungal and bacterial pathogens (Nawrath & Métraux 1999; Wildermuth *et al.*, 2001). The present study is aimed at understanding the hydrogen-bonding networks in the title compound, (I).

The asymmetric unit of title compound (Fig. 1), contains a 2-amino-5-bromopyridinium cation and a 2-hydroxybenzoate anion. In the 2-amino-5-bromopyridinium cation, a wide angle [122.29 (15)°] is subtended at the protonated N1 atom. The 2-amino-5-bromopyridinium cation and 2-hydroxybenzoate anion are essentially planar, with maximum a deviation of 0.020 (1) Å for atom Br1 and 0.018 (2) Å for atom C11, respectively. The anion is stabilized by an intramolecular O1—H1O1···O2 hydrogen bond which generates an S(6) ring motif (Bernstein *et al.*, 1995).

In the crystal packing (Fig. 2), the protonated N1 atom and the 2-amino group (N2) are hydrogen-bonded to the carboxylate oxygen atoms (O3 and O2) *via* intermolecular N1—H1N···O3 and N2—H1N2..O2 hydrogen bonds forming *R*_2_^2^(8) ring motifs. The cation-anion pairs are linked by N2—H2N2···O1 hydrogen bonds into chains propagating along [010]. The crystal structure is further consolidated by π-π interactions between the pyridinium rings at (x,y,z) and (1-x, 1-y, 2-z) [centroid-centroid distance = 3.4908 (10) Å], and that between benzene and pyridinium rings at (x,y,z) and (2-x, 1-y, 2-z), respectively [centroid-centroid distance = 3.5927 (10) Å]. There is a Br1···O3(1-x, 1/2+y, 3/2-z) contact [2.9671 (13) Å] which is shorter than the sum of van *der* Waals radii of the oxygen and bromine atoms.

### Experimental

A hot methanol solution (20 ml) of 2-amino-5-bromopyridine (43 mg, Aldrich) and salicylic acid (34.5 mg, Merck) was mixed and warmed over a magnetic stirrer hotplate for a few minutes. The resulting solution was allowed to cool slowly at room temperature and crystals of the title compound appeared after a few days.

### Refinement

O- and N-bound H atoms were located in a difference Fourier map and allowed to refine freely [O1–H1O1 = 0.89 (3) Å and N–H = 0.82 (2)–0.96 (2) Å]. The remaining H atoms were positioned geometrically and refined using a riding model with C-H = 0.93 Å and *U*_iso_(H) = 1.2 *U*_eq_(C).

### Crystal data

**Table:**

C_5_H_6_BrN_2_^+^·C_7_H_5_O_3_^−^	*F*(000) = 624
*M**_r_* = 311.14	*D*_x_ = 1.709 Mg m^−^^3^
Monoclinic, *P*2_1_/*c*	Mo *K*α radiation, λ = 0.71073 Å
Hall symbol: -P 2ybc	Cell parameters from 6239 reflections
*a* = 8.9498 (2) Å	θ = 2.4–30.1°
*b* = 10.8673 (2) Å	µ = 3.40 mm^−^^1^
*c* = 13.1277 (3) Å	*T* = 100 K
β = 108.704 (1)°	Block, yellow
*V* = 1209.37 (4) Å^3^	0.48 × 0.27 × 0.19 mm
*Z* = 4	

### Data collection

**Table:**

Bruker SMART APEXII CCD area-detector diffractometer	3559 independent reflections
Radiation source: fine-focus sealed tube	2942 reflections with *I* > 2σ(*I*)
graphite	*R*_int_ = 0.023
φ and ω scans	θ_max_ = 30.1°, θ_min_ = 2.4°
Absorption correction: multi-scan (*SADABS*; Bruker, 2009)	*h* = −11→12
*T*_min_ = 0.291, *T*_max_ = 0.572	*k* = −12→15
13299 measured reflections	*l* = −17→18

### Refinement

**Table:**

Refinement on *F*^2^	Primary atom site location: structure-invariant direct methods
Least-squares matrix: full	Secondary atom site location: difference Fourier map
*R*[*F*^2^ > 2σ(*F*^2^)] = 0.025	Hydrogen site location: inferred from neighbouring sites
*wR*(*F*^2^) = 0.070	H atoms treated by a mixture of independent and constrained refinement
*S* = 1.12	*w* = 1/[σ^2^(*F*_o_^2^) + (0.0315*P*)^2^ + 0.5751*P*] where *P* = (*F*_o_^2^ + 2*F*_c_^2^)/3
3559 reflections	(Δ/σ)_max_ = 0.001
179 parameters	Δρ_max_ = 0.62 e Å^−^^3^
0 restraints	Δρ_min_ = −0.48 e Å^−^^3^

### Special details

**Table:**

Experimental. The crystal was placed in the cold stream of an Oxford Cyrosystems Cobra open-flow nitrogen cryostat (Cosier & Glazer, 1986) operating at 100.0 (1) K.
Geometry. All e.s.d.'s (except the e.s.d. in the dihedral angle between two l.s. planes) are estimated using the full covariance matrix. The cell e.s.d.'s are taken into account individually in the estimation of e.s.d.'s in distances, angles and torsion angles; correlations between e.s.d.'s in cell parameters are only used when they are defined by crystal symmetry. An approximate (isotropic) treatment of cell e.s.d.'s is used for estimating e.s.d.'s involving l.s. planes.
Refinement. Refinement of *F*^2^ against ALL reflections. The weighted *R*-factor *wR* and goodness of fit *S* are based on *F*^2^, conventional *R*-factors *R* are based on *F*, with *F* set to zero for negative *F*^2^. The threshold expression of *F*^2^ > σ(*F*^2^) is used only for calculating *R*-factors(gt) *etc*. and is not relevant to the choice of reflections for refinement. *R*-factors based on *F*^2^ are statistically about twice as large as those based on *F*, and *R*- factors based on ALL data will be even larger.

### Fractional atomic coordinates and isotropic or equivalent isotropic displacement parameters (Å^2^)

**Table:**

	*x*	*y*	*z*	*U*_iso_*/*U*_eq_	
O3	0.88434 (14)	0.35437 (12)	0.92381 (10)	0.0225 (3)	
O2	0.98836 (15)	0.30956 (12)	1.09830 (10)	0.0237 (3)	
O1	1.18787 (16)	0.14189 (12)	1.16207 (10)	0.0235 (3)	
C11	1.07156 (18)	0.19199 (15)	0.97440 (13)	0.0161 (3)	
C6	1.1749 (2)	0.12215 (15)	1.05740 (13)	0.0189 (3)	
C10	1.06367 (18)	0.16877 (16)	0.86826 (13)	0.0181 (3)	
H10	0.9957	0.2149	0.8130	0.022*	
C9	1.1556 (2)	0.07793 (17)	0.84408 (14)	0.0215 (3)	
H9	1.1489	0.0625	0.7731	0.026*	
C12	0.97397 (19)	0.29196 (16)	0.99973 (13)	0.0178 (3)	
C8	1.2582 (2)	0.00999 (18)	0.92745 (15)	0.0257 (4)	
H8	1.3207	−0.0507	0.9117	0.031*	
C7	1.2685 (2)	0.03150 (17)	1.03343 (14)	0.0244 (4)	
H7	1.3377	−0.0143	1.0884	0.029*	
H1O1	1.121 (3)	0.204 (3)	1.158 (2)	0.049 (8)*	
Br1	0.370641 (18)	0.746039 (15)	0.758163 (13)	0.01948 (6)	
C5	0.71709 (18)	0.56266 (15)	1.06422 (13)	0.0164 (3)	
C1	0.61778 (19)	0.58819 (16)	0.87434 (13)	0.0178 (3)	
H1	0.6220	0.5662	0.8069	0.021*	
N2	0.81671 (17)	0.50520 (14)	1.14774 (12)	0.0196 (3)	
N1	0.71750 (16)	0.53391 (13)	0.96389 (11)	0.0169 (3)	
C3	0.5063 (2)	0.70608 (16)	0.98575 (14)	0.0191 (3)	
H3	0.4333	0.7637	0.9926	0.023*	
C2	0.51201 (18)	0.67430 (15)	0.88258 (13)	0.0174 (3)	
C4	0.60740 (19)	0.65257 (16)	1.07473 (13)	0.0192 (3)	
H4	0.6050	0.6747	1.1426	0.023*	
H1N	0.784 (3)	0.468 (2)	0.9560 (19)	0.038 (7)*	
H1N2	0.878 (3)	0.452 (2)	1.1347 (17)	0.027 (6)*	
H2N2	0.820 (2)	0.5260 (19)	1.2082 (18)	0.021 (5)*	

### Atomic displacement parameters (Å^2^)

**Table:**

	*U*^11^	*U*^22^	*U*^33^	*U*^12^	*U*^13^	*U*^23^
O3	0.0232 (6)	0.0249 (6)	0.0173 (6)	0.0073 (5)	0.0036 (5)	−0.0019 (5)
O2	0.0309 (6)	0.0257 (7)	0.0153 (6)	0.0064 (5)	0.0085 (5)	−0.0012 (5)
O1	0.0318 (7)	0.0240 (7)	0.0143 (6)	0.0055 (5)	0.0070 (5)	0.0035 (5)
C11	0.0163 (7)	0.0153 (8)	0.0166 (7)	−0.0016 (6)	0.0053 (6)	−0.0021 (6)
C6	0.0236 (8)	0.0165 (8)	0.0166 (8)	−0.0021 (6)	0.0063 (6)	0.0006 (6)
C10	0.0181 (7)	0.0187 (8)	0.0158 (7)	0.0003 (6)	0.0031 (6)	−0.0003 (6)
C9	0.0247 (8)	0.0235 (9)	0.0165 (8)	0.0025 (7)	0.0067 (6)	−0.0026 (7)
C12	0.0173 (7)	0.0184 (7)	0.0175 (8)	−0.0007 (6)	0.0052 (6)	−0.0021 (6)
C8	0.0322 (9)	0.0220 (9)	0.0237 (9)	0.0087 (7)	0.0100 (8)	−0.0008 (7)
C7	0.0307 (9)	0.0210 (9)	0.0196 (8)	0.0077 (7)	0.0054 (7)	0.0048 (7)
Br1	0.02043 (9)	0.01984 (10)	0.01686 (9)	0.00136 (6)	0.00414 (6)	0.00213 (6)
C5	0.0174 (7)	0.0165 (7)	0.0153 (7)	−0.0037 (6)	0.0051 (6)	−0.0022 (6)
C1	0.0194 (7)	0.0205 (8)	0.0140 (7)	−0.0023 (6)	0.0059 (6)	−0.0012 (6)
N2	0.0209 (7)	0.0213 (7)	0.0158 (7)	0.0039 (6)	0.0048 (6)	−0.0009 (6)
N1	0.0184 (6)	0.0178 (7)	0.0145 (6)	0.0003 (5)	0.0054 (5)	−0.0016 (5)
C3	0.0203 (7)	0.0183 (8)	0.0207 (8)	0.0018 (6)	0.0094 (6)	−0.0004 (7)
C2	0.0172 (7)	0.0175 (8)	0.0161 (7)	−0.0002 (6)	0.0035 (6)	0.0010 (6)
C4	0.0216 (7)	0.0193 (8)	0.0175 (8)	−0.0004 (6)	0.0073 (6)	−0.0029 (6)

### Geometric parameters (Å, °)

**Table:**

O3—C12	1.259 (2)	Br1—C2	1.8844 (16)
O2—C12	1.273 (2)	C5—N2	1.327 (2)
O1—C6	1.358 (2)	C5—N1	1.355 (2)
O1—H1O1	0.89 (3)	C5—C4	1.423 (2)
C11—C10	1.395 (2)	C1—C2	1.360 (2)
C11—C6	1.404 (2)	C1—N1	1.361 (2)
C11—C12	1.497 (2)	C1—H1	0.93
C6—C7	1.393 (2)	N2—H1N2	0.86 (2)
C10—C9	1.386 (2)	N2—H2N2	0.82 (2)
C10—H10	0.93	N1—H1N	0.96 (2)
C9—C8	1.393 (2)	C3—C4	1.358 (2)
C9—H9	0.93	C3—C2	1.414 (2)
C8—C7	1.384 (3)	C3—H3	0.93
C8—H8	0.93	C4—H4	0.93
C7—H7	0.93		
			
C6—O1—H1O1	102.4 (18)	N2—C5—N1	118.96 (15)
C10—C11—C6	119.14 (15)	N2—C5—C4	123.01 (15)
C10—C11—C12	120.45 (15)	N1—C5—C4	118.02 (15)
C6—C11—C12	120.40 (15)	C2—C1—N1	120.65 (15)
O1—C6—C7	118.53 (15)	C2—C1—H1	119.7
O1—C6—C11	121.39 (15)	N1—C1—H1	119.7
C7—C6—C11	120.07 (15)	C5—N2—H1N2	117.4 (14)
C9—C10—C11	120.93 (15)	C5—N2—H2N2	118.5 (15)
C9—C10—H10	119.5	H1N2—N2—H2N2	124 (2)
C11—C10—H10	119.5	C5—N1—C1	122.29 (15)
C10—C9—C8	119.19 (16)	C5—N1—H1N	118.3 (14)
C10—C9—H9	120.4	C1—N1—H1N	119.2 (14)
C8—C9—H9	120.4	C4—C3—C2	120.04 (15)
O3—C12—O2	123.71 (16)	C4—C3—H3	120.0
O3—C12—C11	118.98 (15)	C2—C3—H3	120.0
O2—C12—C11	117.31 (15)	C1—C2—C3	118.94 (15)
C7—C8—C9	120.98 (17)	C1—C2—Br1	120.45 (12)
C7—C8—H8	119.5	C3—C2—Br1	120.58 (12)
C9—C8—H8	119.5	C3—C4—C5	120.05 (15)
C8—C7—C6	119.69 (16)	C3—C4—H4	120.0
C8—C7—H7	120.2	C5—C4—H4	120.0
C6—C7—H7	120.2		
			
C10—C11—C6—O1	179.45 (15)	O1—C6—C7—C8	−179.66 (17)
C12—C11—C6—O1	0.9 (2)	C11—C6—C7—C8	−0.5 (3)
C10—C11—C6—C7	0.3 (2)	N2—C5—N1—C1	179.40 (15)
C12—C11—C6—C7	−178.24 (16)	C4—C5—N1—C1	0.2 (2)
C6—C11—C10—C9	0.2 (2)	C2—C1—N1—C5	−0.3 (2)
C12—C11—C10—C9	178.82 (16)	N1—C1—C2—C3	−0.5 (2)
C11—C10—C9—C8	−0.6 (3)	N1—C1—C2—Br1	−178.37 (12)
C10—C11—C12—O3	0.3 (2)	C4—C3—C2—C1	1.2 (3)
C6—C11—C12—O3	178.84 (15)	C4—C3—C2—Br1	179.13 (13)
C10—C11—C12—O2	−179.02 (15)	C2—C3—C4—C5	−1.3 (3)
C6—C11—C12—O2	−0.4 (2)	N2—C5—C4—C3	−178.60 (16)
C10—C9—C8—C7	0.4 (3)	N1—C5—C4—C3	0.5 (2)
C9—C8—C7—C6	0.1 (3)		

### Hydrogen-bond geometry (Å, °)

**Table:**

*D*—H···*A*	*D*—H	H···*A*	*D*···*A*	*D*—H···*A*
O1—H1O1···O2	0.89 (3)	1.66 (3)	2.500 (2)	157 (2)
N1—H1N···O3	0.96 (2)	1.66 (2)	2.611 (2)	172 (2)
N2—H1N2···O2	0.85 (3)	1.98 (2)	2.818 (2)	170 (2)
N2—H2N2···O1^i^	0.82 (2)	2.14 (2)	2.917 (2)	160 (2)

Symmetry codes: (i) −*x*+2, *y*+1/2, −*z*+5/2.
